# Treatment of achalasia led to normalisation of energy metabolism and proteosynthesis dependent on the pool of circulating amino acids; metabolomic monitoring and body composition analysis on insufficient nutrition

**DOI:** 10.1007/s11306-025-02303-6

**Published:** 2025-08-11

**Authors:** Martin Duricek, Eva Baranovicova, Michal Demeter, Diana Vazanova, Lenka Nosakova, Marek Vojtko, Martin Kertys, Jan Lehotsky, Erika Halasova, Peter Banovcin

**Affiliations:** 1 Jessenius Faculty of Medicine in Martin, Clinic of Internal Medicine, Gastroenterology, Comenius University Bratislava, Mala Hora 4, 036 01 Martin, Slovakia; 2 Jessenius Faculty of Medicine in Martin, Biomedical Centre Martin, Comenius University Bratislava, Mala Hora 4, 036 01 Martin, Slovakia; 3 Department of Pharmacology, Jessenius Faculty of Medicine in Martin, Comenius University Bratislava, Mala Hora 4, 036 01 Martin, Slovakia; 4 Department of Medical Biochemistry, Jessenius Faculty of Medicine in Martin, Comenius University Bratislava, Mala Hora 4, 036 01 Martin, Slovakia

**Keywords:** Achalasia, POEM, Energy metabolism, Diet, Protein synthesis, Plasma metabolomics

## Abstract

**Introduction:**

Achalasia is a rare motility disorder of the esophagus characterized by the loss of the propulsive peristalsis and impaired relaxation of the lower esophageal sphincter. Patients with untreated achalasia suffer from dysphagia and regurgitation and, consequently, weight loss as they require dietary modifications related to impaired esophageal emptying. Peroral endoscopic myotomy (POEM) is considered the mainstay of therapy, leading to symptom relief, restoration of normal eating patterns and weight gain.

**Objectives:**

This study aims to describe the metabolic state of an organism in time of insufficient nutrition and the relation among levels of systemic metabolites and changes in body’s composition in condition of normalised food intake.

**Methods:**

We monitored body composition, as well as biochemical parameters in 43 patients with achalasia before and 3 months after POEM intervention. We also determined the levels of circulating metabolites via NMR spectroscopy which were, besides comparison to controls, used to describe metabolic turnover before and after treatment.

**Results:**

After POEM, all patients except four individuals gained weight (p-value 3.07e-11), and accordingly, the BMI value changed (p-value 2.85e-9). Paired test revealed an increase in absolute fat (p-value 0.00176) and muscle mass (p-value 0.00201). Metabolic analysis in patients with untreated achalasia showed a ketotic-like state with inadequate glycolysis and gluconeogenesis, which partially normalized three months after POEM. Post-POEM muscle mass gain was positively (*p* < 0.05-0.0005) and increase in absolute fat mass was negatively correlated (p-value < 0.05-0.0005) with the levels of circulating amino acids before the intervention.

**Conclusion:**

Our observations provide complex insight into the metabolic shifts after successful treatment of achalasia that is directly related to the changes in the body composition. The metabolic alterations were not detectable through standard biochemical tests suggesting that conventional diagnostics may not fully reflect the metabolic condition of patients with achalasia.

## Introduction

**Fig. 1:**
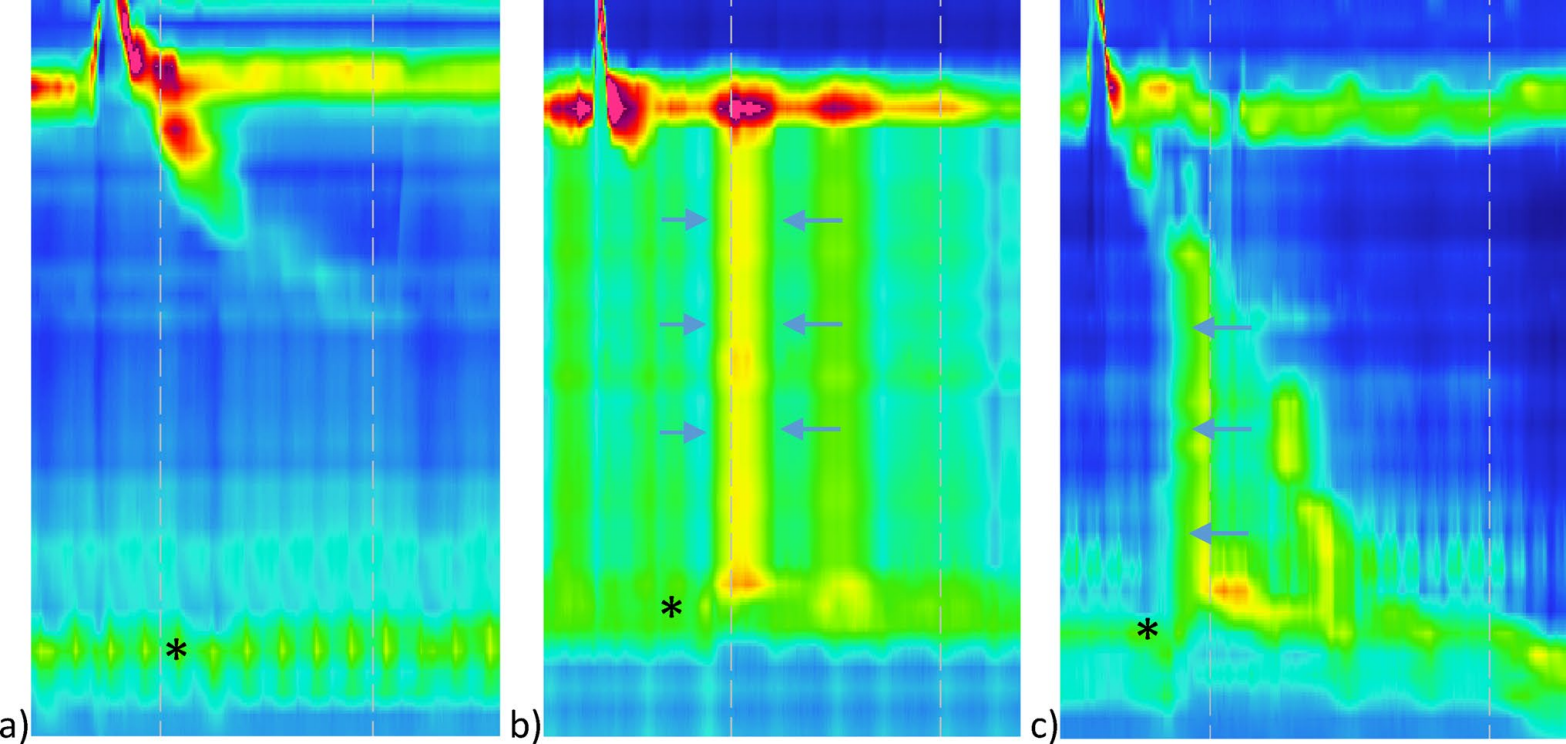
Subtypes of achalasia according to the high resolution manometry. **a** Type I achalasia represented by the impaired relaxation of the lower esophageal sphincter (LES) (*). **b** type II achalasia represented by the impaired relaxation of the LES (*) and panesophageal pressurizations (blue arrows). **c** Type III achalasia represented by the impaired relaxation of the LES (*) and premature contractions (blue arrows)

Achalasia is a primary esophageal motility disorder that is characterized by the absence of esophageal propulsive peristalsis and impaired relaxation of the lower esophageal sphincter. Patients with achalasia experience dysphagia, regurgitation and non-cardiac chest pain and consequently suffer from inadequate caloric intake due to adjustment of their diet to impaired esophageal emptying. High resolution esophageal manometry is regarded as the diagnostic golden standard, and based on this, 3 subtypes of achalasia are distinguished (“Esophageal motility disorders on high-resolution manometry: Chicago classification version 4.0© - Yadlapati − 2021 - Neurogastroenterology & Motility - Wiley Online Library” n.d.). Type I is defined by impaired relaxation of the lower esophageal sphincter (LES) and no panesophageal pressurization. This likely represents long-lasting achalasia as dilation of esophageal lumen is quite common. Type II has the features of panesophageal pressurizations and type III is represented by premature contractions (distal latency < 4.5s) (Fig. 1).

Although the diet composition remains unique in the individual patient, weight loss is a common unifying feature that occurs in a substantial proportion of achalasia patients. Peroral endoscopic myotomy (POEM) has gained acceptance as the standard of therapy (Weusten et al., 2020) that provides long-term symptom relief in 87% of patients (Vespa et al., 2023). After POEM intervention, patients do not usually require any dietary restrictions and provided their symptoms are resolved, the vast majority of individuals gain weight, which is a result well-appreciated by clinicians.

So far, a relatively small number of studies have been performed on documenting the details of general weight gain. One single-center study retrospectively analyzed BMI and observed weight gain in general, with an increase in the proportion of pre-obese and obese patients (Hew et al., 2021). Another study went further with the use of dual bioelectrical impedance analysis to determine the body composition analysis that revealed an increase of visceral and subcutaneous fat volumes with a less prominent, still significant increase in the skeletal muscle volume (Mizusawa et al., 2020).

In patients with achalasia that undergo POEM, weight gain is only one of many and very macroscopic indicator for describing the conditions after the increased diet intake in the post-intervention period. The improved nourishment influences the reservoirs of energy sources, essential amino acids and other nutrients that could be altered in time of inadequate food supply. Although the nutrition, metabolism and weight gain are closely tied, we are not aware of detailed metabolic studies on individuals linked with the increase in body mass and changes in body’s composition. Metabolic alterations in subjects suffering from anorexia nervosa (AN), that are critically underweight (body mass index around 15) were reviewed by Martinez et al. (“Unveiling Metabolic Phenotype Alterations in Anorexia Nervosa through Metabolomics” n.d.) but the data lack a link to the body composition. More research effort has been focused lately on weight loss (type 2 diabetes mellitus) (“Unveiling Metabolic Phenotype Alterations in Anorexia Nervosa through Metabolomics” n.d.) and on obese individuals after a period of intensive exercise (Lin et al., 2023) and after gastric bypass surgery (“Characterization of the plasma metabolome and lipidome in response to sleeve gastrectomy and gastric bypass surgeries reveals molecular patterns of surgical weight loss - ScienceDirect” n.d.). Although changes in phosphatidylcholines and other lipid species are reported, there are limited data on the changes in skeletal muscle mass and body fat.

In our study, the first of its kind, we monitored the conditions of patients with achalasia at two time points–before and 3 months after the POEM intervention. We performed an in-depth analysis of the body composition using a bioimpedance scale supplemented by the state-of-the-art metabolomics analysis of blood plasma samples and also standard biochemical analysis to shed light on the shift of biochemical processes after the reversal of the eating pattern in patients with achalasia undergoing peroral endoscopic myotomy. The metabolic data were compared in patients before and after POEM and also together with the matching sample from the general population to show the differences in the functioning of energy metabolism and amino acids reservoirs. Additionally, we sought the association between pools of circulating amino acids and body composition changes 3 months post POEM, which could reveal important relations useful in understanding the generalised body’s response when recovering from the conditions of dietary insufficiency.

## Methods

This was a prospective single-center study. Consecutive patients with esophageal achalasia referred to the Clinic of Internal Medicine– Gastroenterology between August 2020 and August 2022 that have been confirmed by high-resolution manometry (HRM) were prospectively screened for eligibility. High resolution manometry measurements were performed while outpatient in our institution (ManoScan™ ESO High Resolution Manometry System, Medtronic, USA). Inclusion criterion was symptomatic achalasia with duration of symptoms min. 3 months, Eckardt score greater than two (represents the indication for POEM), which was yet untreated, and POEM was the first intervention. Exclusion criteria were age below 18 years old, alcohol consumption > 40 g/d, history of surgery affecting the esophagus, stomach and/or esophagogastric junction, bowel resections, pregnancy or breastfeeding, neurological disorders, active malignancy, advanced chronic liver disease, an active inflammatory bowel disease that might otherwise interfere with the nutritional status of the patient, eating disorders and avoidant/restrictive food intake disorder. Written informed consent was obtained from each eligible subject who agreed to be enrolled in the study.

### Data collection while inpatient

A total of 43 patients with achalasia were included in the final analysis (22 M/21F). The median age was 45 years, with an interquartile range (IQR) of 8 years. There were 16 patients with type I achalasia, 19 patients with type II achalasia, one patient with type III achalasia. In the remaining 7 achalasia patients the HRM catheter could not have been passed through the lower esophageal sphincter due to the dilation of the esophageal lumen. In these cases no propulsive peristalsis was present on the HRM recording and the diagnosis was confirmed by the barium swallow study. The duration of symptoms was available in 36 patients. The median duration of symptoms was 15 [9–24] months. Importantly, POEM led to significant improvement of symptoms (Eckardt score 6.88 ± 1.9 vs. 0.71 ± 0.76 before and 3 months after the procedure, resp.). Only one patient had Eckardt score > 2 after the intervention.

The first part of the study was performed on an inpatient basis while the patient was admitted due to the POEM procedure. Before POEM, a careful interview was conducted to assess for achalasia symptoms like dysphagia, regurgitation, non-cardiac chest pain and weight loss. These were semi-quantitatively evaluated, and the Eckardt score was calculated as part of a routine clinical investigation (Taft et al., 2018). Blood samples were collected in the morning in the fasted state for the following haematological and biochemical analysis: differential blood count, glucose, triglycerides, LDL and HDL cholesterol, albumin, total protein count, transferrin levels, creatinine, urea, TSH (thyroid stimulating hormone), vitamin D and B12 levels. These were chosen to reflect the nutritional status of the patients. At the same time, blood was also collected for the metabolomic analysis (see below). The same day before POEM (still fasting), patients were evaluated by the bioimpedance scale (Tanita MC-780 MA, Japan). The following parameters were obtained: total weight, the mass of fat and muscle, the relative mass of fat and muscle (in % related to whole body mass), and BMI was calculated. Waist girth, hipline, arm circumferences and skinfold on the dominant arm were manually measured using a tape measure and skinfold caliper and waist-to-hip ratio was calculated.

POEM procedure was performed under the general anesthesia in the standard manner that was described elsewhere (Inoue et al., 2010). We used Olympus-HQ190 (Olympus, Tokyo, Japan) with a transparent cap (Fuji-film DH-28GR) and an electrosurgical unit (VIO ERBE 300D). A triangular tip knife (TT-knife KD-640 L, Olympus, Tokyo, Japan) was used for the mucosal entry, tunneling of submucosa after the instillation of submucosal space with 0.2% indigo carmine in normal saline and myotomy. The mucosal entry was closed by hemoclips (EZ clip Olympus HX-610–135 L, Resolution 360° clip Boston Scientific, Marlborough, MA, USA). Before and after the procedure, the patients received three doses of broad-spectrum antibiotics as prophylaxis (metronidazole, cefuroxime). Patients were discharged after gaining the ability to tolerate a semi-liquid diet. Patients were routinely prescribed proton pump inhibitors (PPI) twice daily for 2 weeks after discharge, followed by the dose reduction to once daily (morning dose) until the date of outpatient check up to prevent gastroesophageal reflux.

Some patients had internal comorbidities, of which the most prevalent was arterial hypertension (44% of patients), diabetes mellitus and/or impaired fasting glucose (9% of patients), coronary artery disease or cerebral arteriosclerosis (12% of patients), and chronic kidney disease (G2) with normal creatinine level (1 patient). Autoimmune thyroiditis was present in 26% of patients; however, all of the patients had TSH levels within the normal range. One patient had celiac disease with serological and histological remission on a gluten-free diet. Fatty liver disease was present in 16% of patients. 23% of patients were diagnosed with dyslipidemia according to their medical records; however, none of the patients had a high LDL level (> 4.5 mmol/l), and only two patients had a high total cholesterol level (> 6.5 mmol/l). Importantly, no patient had any newly diagnosed comorbidity or experienced any significant alteration of their state of health between the 1st and 2nd visit.

After three months, the second part of the study was conducted– patients were invited for outpatient checkups. During the checkup, symptoms in general and Eckardt score were evaluated, fasting blood samples were collected, and bioimpedance scale measurements were performed as described above. Changes in the chronic medication (apart from PPI that was routinely prescribed to prevent acidic gastroesophageal reflux post-POEM) were verified.

### Controls

Blood samples from 43 subjects (22 M/21F), age median 43, IQR 7 years, representing the normal population were selected in order to achieve a comparable group with agreement in BMI (comparable to the post-POEM state). Control individuals reported being subjectively healthy and not being aware of any acute or chronic illnesses. All of the healthy subjects reported themselves to be without any gastrointestinal diseases or disorders, neither they were taking any medication involving the effect on gastrointestinal function, nor they were investigated by gastroenterologist specialist.

### Blood handling for metabolomic measurements

Blood was collected in EDTA-coated tubes and centrifuged within 1 h after collection to plasma at 4 °C, 2000 rpm (380 g-force) for 20 min. The samples were stored at − 80 °C until use. Plasma denaturation was carried out according to Gowda et al.(Nagana Gowda et al., 2015): 600 µL of methanol was added to 300 µL of blood plasma, the mixture was vortexed and frozen at − 24 °C for 20 min. After subsequent centrifugation at 14 000 rpm (14 800 g-force) for 30 min, 700 µL of supernatant was taken, dried, and stored at − 80 °C. Before NMR measurement, the dried matter was solved in 500 µL of deuterated water and 100 µL of stock solution (phosphate buffer 200 mM, pH 7.4– pH meter reading), 0.30 mM TSP-d_4_ (trimethylsilylpropionic acid-d_4_) as a chemical shift reference in deuterated water). Finally, the final solution was transferred into a 5 mm NMR tube.

### Data acquisition and processing

NMR data were acquired on 600 MHz NMR spectrometer Avance III from Bruker equipped with TCI CryoProbe at T = 310 K. Initial settings (field shimming, receiver gain, water suppression frequency) were carried out on an independent sample and adopted for measurements. After preparation, samples were stored in a Sample Jet automatic machine for not more than 2 h and cooled at approximately 5 °C. Before measurement, each sample was preheated on the 310 K for 5 min. An exponential noise filter was used to introduce 0.3 Hz line broadening before the Fourier transform. All data were once zero-filled. Samples were randomly ordered for acquisition.

Bruker profiling protocols were modified as follows: noesy with presaturation (noesygppr1d): FID size 64k, dummy scans 4, number of scans: 64, spectral width 20.4750 ppm; profiling cpmg (cpmgpr1d, L4 = 126, d20 = 3 ms): number of scans: 256, spectral width 20.4750 ppm. For 15 randomly chosen samples, 2D spectra were measured: cosy with presaturation (cosygpprqf): FID size 4 k, dummy scans 8, number of scans 16, spectral width 16.0125 ppm; homonuclear J-resolved (jresgpprqf): FID size 8 k, dummy scans 16, number of scans 32. All experiments were conducted with a relaxation delay of 4 s. For all samples, we kept the half-width of the TSP-d_4_ signal under 1.0 Hz.

Spectra were solved using a human metabolomics database (www.hmda.ca) (Wishart et al., 2022), Chenomx software (free trial version), an internal metabolite database, and by researching metabolomics literature (Nagana Gowda et al., 2015). The proton chemical shifts are reported relative to the TSP-d_4_ signal assigned a chemical shift of 0.000 ppm. The peak multiplicities were confirmed in J-resolved spectra, and homonuclear cross-peaks were confirmed in 2D cosy spectra. All spectra were binned in bins of the size of 0.001 ppm. No further normalization method was applied to the data, as we worked with exactly the same amount of blood plasma for measurements. Then, intensities of selected bins were summed only for spectra subregions with only one metabolite assigned or minimally affected by other co-metabolites. Metabolites showing weak intensive peaks or strong peak overlap were excluded from the evaluation. The obtained values were used as relative concentrations of metabolites in a sample.

### Data analysis

Shapiro–Wilk test rejected normality in approximatelly half of the data. The null hypothesis of equality of population medians among patients before and after POEM as well as in comparison to controls was tested by the non-parametric Mann-Whitney U test. The null hypothesis that the median of the population of differences between the paired data before and after POEM is zero (i.e. whether there is a difference between paired observations) was tested using the Paired Wilcoxon Signed rank test for paired samples. To test for linear associations among data, we used Pearson’s correlation test. Calculations were performed in OriginPro 2018b (v.9.5, OriginLab, USA) and Matlab (v. 2015b, Mathworks, USA). After Bonferroni correction to avoid Type I error (risk of obtaining false-positive results when multiple tests are performed on a single set of data), p-value of 0.002 was used as a threshold to claim significance.

### Note

In this work we use the trivial names of 2-oxoisocaproate– ketoleucine, 3-methyl-2-oxovalerate-ketoisoleucine and 2-oxoisovalerate– ketovaline to better evoke the origin of the ketoacids.

## Results

### Routine biochemistry data

After POEM, there was a significant increase in the level of blood glucose, cholesterol, HDL, as well as albumin level and total protein count and decrease of the creatinine level without a corresponding decrease of the blood urea level. Also, increase in the TSH and an increase in vitamines D and B12 of the marginal significance was observed. Patient data from routine biochemical examinations are summarized in Table 1.

**Table 1:** Biochemical parameters related to the status of nutrition before and after the POEM intervention

	Before POEM	After POEM	*p*-value	Reference values
Glucose (mmol.l^− 1^)	4.4 [4–5]	5.3 [4.6-5.8]	2.98e-4	4.1–5.9
Cholesterol (mmol.l^− 1^)	4.78 [4.18–5.34]	4.96 [4.47-6]	0.00123	elevated > 5.17
TAG (mmol.l^− 1^)	1.01 [0.77-1.62]	0.96 [0.7–1.45]	0.7194	0.4–1.7
HDL (mmol.l^− 1^)	1.39 [1.16–1.66]	1.82 [1.54–2.2]	2.942e-5	1.03–2
LDL (mmol.l^− 1^)	3.39 [2.51–3.67]	3.22 [2.24–3.79]	0.72772	1–3.3
Albumin (g.l^− 1^)	41.65 [39.5–44.1]	43.55 [41.8–45.4]	0.00853	35–52
Total protein count (g.l^− 1^)	71.3 [68.2–74.2]	74.8 [72.58–77.73]	5.0019e-5	66–83
Transferrin (g.l^− 1^)	2.16 [1.95–2.49]	2.57 [2.37–2.77]	3.95886e-7	2–3.6
TSH (mU.l^− 1^)	1.29 [0.83–1.87]	1.76 [1.29–2.83]	0.04329	0.38–5.33
vit. D (ug.l^− 1^)	30.8 [20.95–33.75]	31.5 [24.7–36]	0.25129	30–100
B12 (pmol.l^− 1^)	86.1 [66.3-126.05]	88.2 [53.93-116.58]	0.16707	25.1-165
Creatinine (µmol.l^− 1^)	74 [62.25–85.5]	71.5 [62–80]	0.02497	59–104
Urea (mmol.l^− 1^)	4.3 [3.78–5.73]	4.6 [3.7–5.38]	0.40466	2.8–7.2

Data are expressed as median with the Q1 and Q3 ranges

P values derived from paired sample Wilcoxon signed rank test

### Metabolic data handling

For statistical evaluation of metabolic changes in patients before and after POEM, we used both paired Wilcoxon signed-rank test for pairwise comparison and the Mann-Whitney U test for testing of the equality of medians between groups. The relative blood plasma levels of branched chain amino acids (BCAAs): leucine, isoleucine and valine, and branched chain keto acids (BCKAs): ketoleucine, ketoisoleucine and ketovaline showed a common pattern: decreased plasma levels after POEM intervention. Three months after POEM, patients showed also statistically significant elevation of blood glucose levels accompanied by a decrease in circulating 3-hydroxybutyrate, an increase in blood plasma level of alanine and tryptophan. For comparison to control group, Mann Whitney U test was employed. Patients showed significantly decreased relative levels of glucose, glutamine, alanine, creatine, tryptophan and histidine, and increased levels of 3-hydroxybutyrate at both sampling times. The results are summarized in detail in Table 2.

When we compared the values of plasma glucose levels from clinical biochemistry and NMR measurements, Pearson’s correlation resulted in *r* = 0.901 with a p-value of 9.4e-16 (for 43 samples). It should be noted that clinical values are discrete values rounded to first decimal place, while values obtained from NMR are continuous data. In light of this, we consider this result a good match between the two data sets.

**Table 2:** Statistical comparison of blood metabolites in patients before POEM, after POEM and controls, % change derived from medians

	before/after POEM	before/after POEM		before POEM/ctrl		after POEM/ctrl	
	Paired Wilcoxon signed rank test	Mann - Whitney U test		Mann - Whitney U test		Mann - Whitney U test	
	p-value	p-value	% change	p-value	% change	p-value	% change
Lactate	n.s.	n.s.	x	2.31e-08	– 33	1.42e-08	– 34
Alanine	1.21e-04	3.73e-03	– 18	2.08e-17	– 34	6.78e-12	– 20
Valine	6.70e-04	2.03e-03	12	n.s.	x	1.45e-08	– 19
Glucose	6.36e-08	8.93e-06	– 11	1.73e-08	– 18	0.02738	– 7
Leucine	1.74e-06	4.22e-06	23	n.s.	x	4.49e-10	– 22
Isoleucine	1.18e-05	3.37e-05	28	n.s.	x	8.01e-09	– 22
Pyruvate	n.s.	n.s.	x	4.67e-04	– 16	2.34e-05	– 21
Citrate	0.0082	n.s.	x	0.00151	10	n.s.	x
Phenylalanine	0.04314	n.s.	x	3.87e-07	– 21	1.10e-12	– 22
Tyrosine	n.s.	n.s.	x	1.69e-12	– 31	8.99e-14	– 25
Glutamine	n.s.	n.s.	x	6.08e-07	– 16	1.91e-05	– 15
Lysine	n.s.	n.s.	x	9.93e-07	– 13	7.14e-08	– 14
3-Hydroxy-butyrate	1.93e-07	9.38e08	142	2.96e-11	273	0.02564	12
Ketoleucine	2.94e-04	7.05e-03	15	n.s.	x	n.s.	x
Ketoisoleucine	0.00466	0.02798	10	n.s.	x	0.04618	– 7
Ketovaline	0.048053	0.04714	x	n.s.	x	n.s.	x
Creatine	n.s.	n.s.	x	8.93e-06	– 38	1.15e-04	– 34
Creatinine	0.00274	n.s.	x	n.s.	x	n.s.	x
Proline	0.02092	n.s.	x	1.16e-14	– 32	5.50e-10	– 30
Histidine	0.01866	n.s.	x	6.03e-06	– 13	7.21e-11	– 12
Tryptophan	1.04e-04	3.69e-04	– 13	1.74e-15	– 34	1.83e-10	– 24
Succinate	1.61e-04	8.22e-05	31	1.44e-07	51	0.04006	15

Listed p-values below 0.05, after bonferroni correction was the Treshold to claim significance set to 0.002, n.s. are markerd tests with a p-value > 0.05

### Body composition analysis data based on the bioimpedance scale

**Fig. 2:**
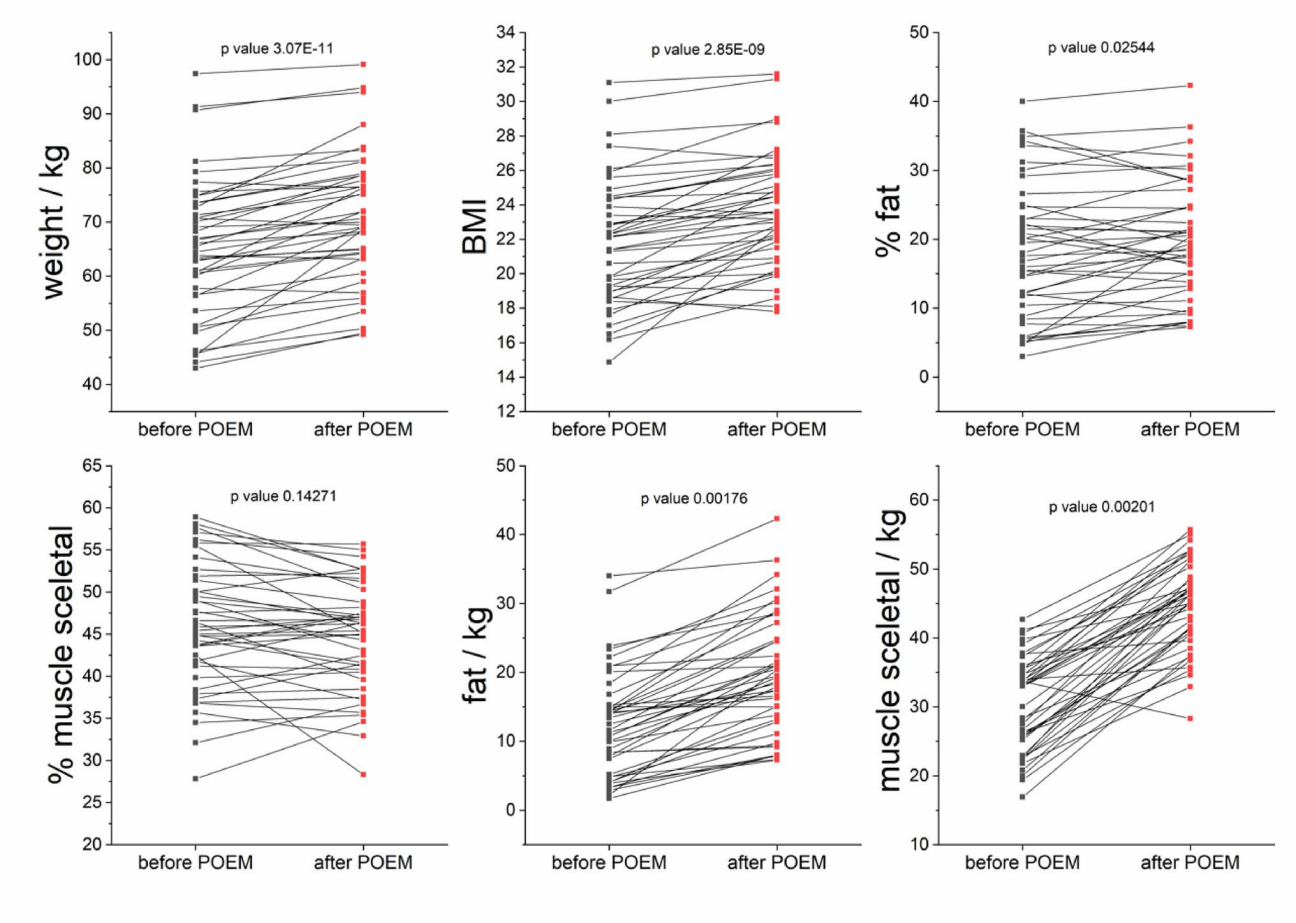
Patients’ bioimpedance data before/after POEM, pairwise comparison, p-values derived from paired Wilcoxon signed rank test, % fat/muscle represents a proportion of fat/muscle in the body

After the POEM intervention, the patients’ body mass and composition significantly changed. Before POEM, 7 patients (16.3%) were underweight (BMI < 18.5), 6 (14%) were overweight (BMI > 25) and 1 patient (2.3%) was obese (BMI > 30). 29 patients were of normal weight (BMI 18.5–25). After POEM, 2 patients (4.6%) were underweight, 12 patients (27.9%) were overweight and 2 patients were obese (BMI > 30). 27 patients were of normal weight. All patients except four gained weight, and the BMI values changed accordingly. In another four patients, we observed reduced waist circumference. Except for one patient, hip circumference increased in all patients. Skinfold thickness decreased in 16, did not change in 8 and increased in 19 patients. 15 patients showed decrease and 28 increase in % fat. 23 patients lost, and 20 patients gained relative muscle weight. Absolute muscle weight in kg was gained in 11 patients, in 23 patients the weight was lost. Absolute fat weight in kg was gained in 30 patients, and lost in. The comparison of bioimpedance data in patients before/after POEM is shown in Table 3; Fig. 2.

**Table 3:** Comparison of body’ parameters/composition in patients with achalasia before and after POEM intervention, p-value derived from pairwise comparison, BMI - body mass index, WHR - waist/hip ratio

	Before POEM	After POEM	
	Median (IQR)	Median (IQR)	p-value
Weight/kg	65.5 (16.9)	70(15.3)	3.07e-11
BMI	22.1(5.3)	23.5(4.5)	2.85e-09
Waist circumference /cm	80(16.5)	85(17)	1.73e-07
WHR	0.87(0.09)	0.88(0.13)	0.63551
Skinfold/cm	11(7)	14(9)	0.0029
% fat	17.6(14.3)	19.3(11.6)	0.02544
% muscle	45.4(10.2)	45.4(8.3)	0.14271
kg fat	11.6(10.1)	13.7(10.8)	0.00176
kg muscle	33(10.2)	32.6(11.5)	0.00201

Having patients’ data about the post-POEM changes in body fat and muscle amount, it was of interest if the patients’ metabolic data before intervention relate to post-operation changes. We used Pearson‘s correlation to test the relationship between relative levels of circulating metabolites before POEM and changes in body composition within the timeframe from the POEM intervention to the follow-up examination. We obtained a statistically significant relationship between pools of circulating amino acids and differences in body fat and muscle proportions before and after POEM (Table 4). No relationships were found for other metabolites. We compared the weight gain and BMI change between achalasia subtypes I and II (having a sufficient high sample size) and these were not significantly different from each other. Based on this evidence and the fact that we are in our manuscript focused on metabolic changes related to changes in body mass, we did not include separate data evaluations for the achalasia subtypes.

**Table 4:** Pearson correlation of relative blood plasma amino acids levels before POEM with changes in fat and muscles after POEM intervention

	% fat delta		% muscle delta		kg fat delta	
	Pearson Corr.	p-value	Pearson Corr.	p-value	Pearson Corr.	p-value
Valine	– 0.61321	1.23e-05	0.5166	3.90e-04	– 0.57431	5.65e-05
Leucine	– 0.58787	3.40e-05	0.49401	7.61e-04	– 0.53252	2.37e-04
Isoleucine	– 0.53484	2.20e-04	0.45245	0.00232	– 0.48551	9.66e-04
Tyrosine	– 0.41147	0.00612	0.33992	0.02574	– 0.37129	0.01424
Glutamine	– 0.3277	0.03194	0.27556	0.07369	– 0.38701	0.01035
Lysine	– 0.38755	0.01024	0.25583	0.09776	– 0.46781	0.00156
Ketoleucine	– 0.45448	0.0022	0.4071	0.00674	– 0.43057	0.00395
Ketoisoleucine	– 0.43973	0.00317	0.41839	0.00524	– 0.40831	0.00656
Ketovaline	– 0.48872	8.83e-04	0.39782	0.00825	– 0.44457	0.00282
Histidine	– 0.46457	0.0017	0.42335	0.00468	– 0.47062	0.00145

## Discussion

**Fig. 3:**
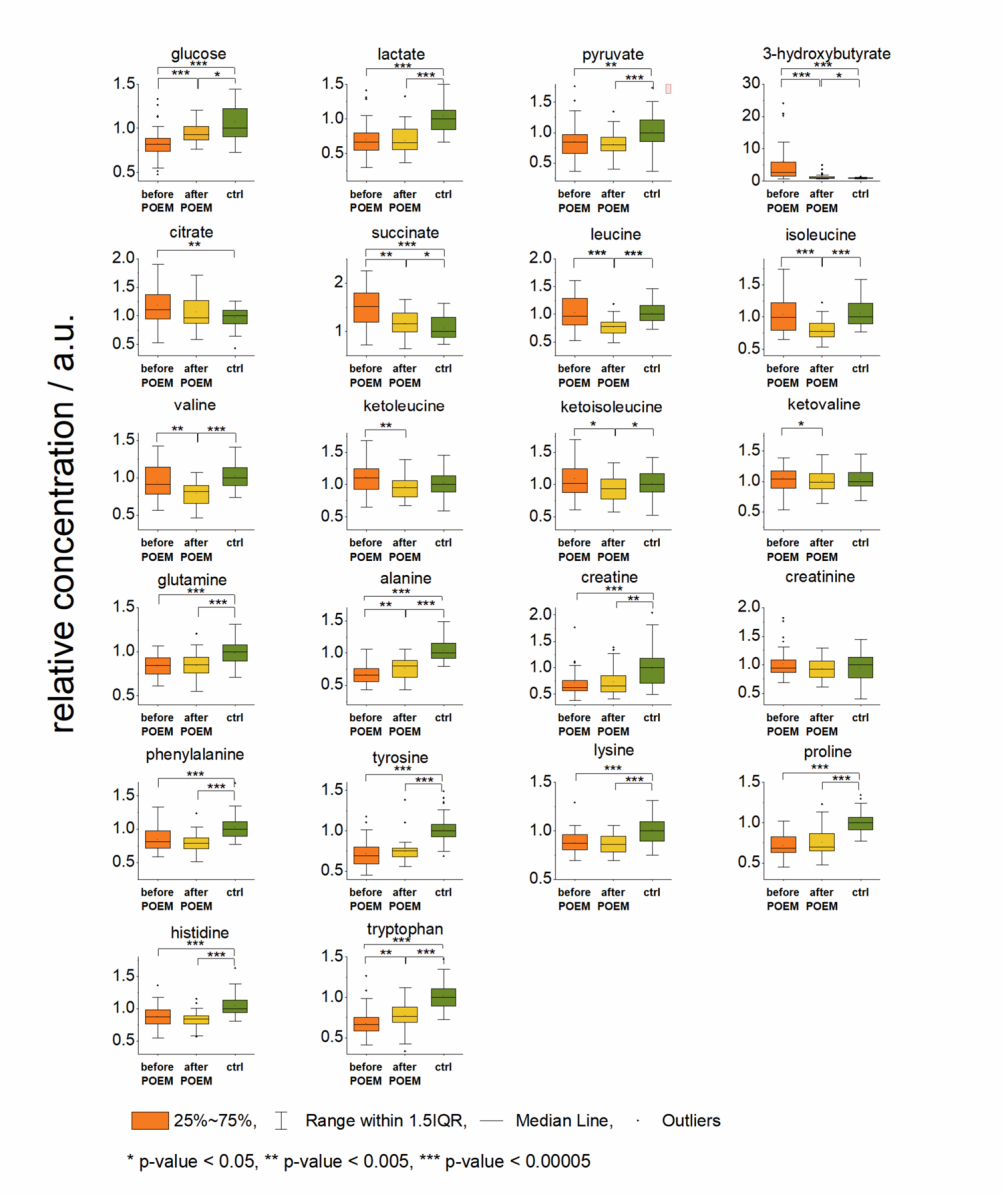
Relative levels in blood plasma metabolites involved in energy metabolism and amino acids in patients before and after POEM and controls, data normalized to medians of controls set to 1, p-values derived from Mann Whitney U test

Glucose is a primary energy source in mammals, including humans, and blood glucose levels are one of the fundamental clinical parameters used to assess the functioning of energy metabolism. In our study, achalasia patients before POEM showed significantly lower blood glucose levels compared to controls. Three months after the POEM intervention, blood glucose levels increased; however, they still remained lower than those measured in healthy subjects (Fig. 3). The glucose levels observed in patients were biochemically within the physiological range and did not lead to clinically relevant hypoglycaemia, however indicated lowered glucose availability in the circulation. A limited number of studies focused on metabolic changes in time of weight-gaining. Data from critically underweight (BMI ~ 15) patients suffering from anorexia nervosa showed decreased glucose levels in acute phase, which are still present in recovered patients when compared with controls (Salehi M. et al. 2021). Glucose availability and the functionality of additional mechanisms necessary for glucose utilization are crucial factors for the formation of lactate and pyruvate. During periods of reduced glucose accessibility, pyruvate and lactate production is limited, as manifested by the reduced abundances of both metabolites in the blood plasma of patients with untreated achalasia (Fig. 3).

Blood lactate has gained special attention over the recent years as it was revealed that the contribution of glucose to the TCA (tricarboxylic acid) cycle is indirect, and the circulating lactate is a primary circulating substrate for the TCA cycle in most tissues (Hui et al., 2017; Rabinowitz & Enerbäck, 2020). In patients with achalasia included in this study, the levels of TCA intermediates succinate and citrate were increased in blood plasma (Fig. 3). Accumulation of citrate and succinate may indicate that the TCA cycle is not operating at full capacity, potentially reducing its efficiency, discussed below in detail.

During the prolonged period of reduced glucose levels in circulation (represented in this study by patients with untreated achalasia), hepatic gluconeogenesis plays a crucial role in maintaining glucose homeostasis to meet the energy demands of extrahepatic tissue and the entire organism. This process is regulated on multiple levels (Zhang et al., 2019). One of the major glucogenic precursors, and the vehicle for interorgan transport of protein-derived carbon and nitrogen through plasma for glucose carbon skeleton is glutamine (Nurjhan et al., 1995; Oliveira-Yamashita et al., 2009). Besides this, circulating metabolites lactate and alanine substantially contribute to the *de novo* synthesis of glucose in the liver, thereby participating in pyruvate production via the Cori and Cahill cycles (Katz & Tayek, 1998; Sarabhai & Roden, 2019). Patients with untreated achalasia showed lowered blood levels of all three primary glucogenic blood plasma metabolites: glutamine, lactate and alanine compared to controls, still persisting three months after POEM. Together with lowered blood glucose levels, these results suggest diminished gluconeogenesis in patients with untreated achalasia, somewhat improved but still persisting in the same patients after the intervention and improved intake of normal diet (Fig. 3).

When glycolysis does not satisfy the body’s energy requirements, alternative energy substrates such as ketone bodies could support metabolic demands (Puchalska & Crawford, 2017). Indeed, we observed an almost four times higher amount of 3-hydroxybutyrate in blood plasma in patients with untreated achalasia relative to controls, remaining still increased, however, to a lower extent, three months after POEM (Fig. 3). In time of impaired glycolysis, 3-hydroxybutyrate can supply up to 50% of the basal energy requirements for highly oxidative tissues and provide as much as 70% of the brain’s energy needs, even more efficiently than glucose (White & Venkatesh, 2011). 3-hydroxybutyrate is a representative of ketone bodies produced mainly by hepatocytes. However, local ketogenesis can also occur in other cells (e.g., gut epithelial cells, astrocytes, kidneys, pancreatic beta cells) (Puchalska & Crawford, 2017). The autocrine effect of local ketogenesis allows cells to adapt to changes in their energy demands and metabolic environment, ensuring efficient functioning during periods of fasting or altered nutrient availability. Non-hepatic ketone production can reduce lactate production in tissues that typically rely on glycolysis and lactate-pyruvate balance may become altered. In tissues like the liver, where ketogenesis is prominent, acetyl-CoA from fatty acid oxidation not only fuels ketone production, but can also enter the TCA cycle to generate ATP. However, during fasting or low-carbohydrate conditions, oxaloacetate availability becomes a limiting factor as oxaloacetate is also used in gluconeogenesis. Low oxaloacetate impairs the TCA cycle, as acetyl-CoA cannot combine with oxaloacetate to form citrate, halting the cycle. Consequently, excess acetyl-CoA is diverted into ketogenesis, and intermediates like citrate and succinate accumulate, indicating a less efficient or incomplete TCA cycle.

Besides their involvement in gluconeogenesis, alanine and glutamine also play an essential role in the ammonia detoxification of extrahepatic tissues and significantly contribute to the interorgan ammonia exchange (Levitt & Levitt, 2018).Their production is ensured by the catabolism of BCAAs (branched chain amino acids) mostly in muscles, which involves the reversible transfer of the BCAA amino group to alpha-ketoglutarate to form corresponding ketoacids – BCKAs (branched chain keto acids) and glutamate. The direction of reverse BCAAs transamination ought to respond to the changes in the concentrations of BCAAs and BCKAs and the availability of the donors and acceptors of nitrogen. Glutamate acts as the source of the amino group to form alanine from pyruvate or as a substrate for ammonia detoxification to glutamine. Glutamine, alanine, and a significant portion of the BCKAs are released from muscles into the bloodstream. The second catabolic step of BCKAs, irreversible decarboxylation to the corresponding branched-chain acyl-CoA esters, occurs mainly in the liver and kidneys and to a lower extent in other tissues (Harper et al., 1984). In this way, BCAAs contribute to energy-gaining metabolic pathways and, in addition to that, also indirectly modulate glucose metabolism. Taking into consideration that leucine is ketogenic, isoleucine is both ketogenic and glucogenic and valine is primarily glucogenic, the impact of BCAAs on energy metabolism may be diverse. In our study, due to the suspected undernutrition in patients with untreated achalasia, the all three BCAAs and BCKAs levels showed values comparable to those found in control subjects. According to the results observed, the contribution of BCAAs to energy metabolism (via intermediates entering the TCA cycle) seems not to be significant in these subjects, indicating balanced nitrogen metabolism as also seen in urea levels. However, other aspects of circulating BCAAs levels are discussed below.

Unlike BCAAs, most of the other amino acids are metabolized in the liver via cytosolic aminotransferase to glutamate, which is then converted to urea that binds excessive ammonia from an organism. Patients with untreated achalasia showed normal blood urea values both before and after the intervention, indicating no signs of excessive muscle breakdown or increased catabolism, as well as good renal health. The intact kidney functioning in patients before POEM can also be derived from normal ranged creatinine values (Table 1), which were slightly, in pairwise test significantly, decreased after the POEM intervention, suggesting somewhat improved creatinine clearance after POEM.

POEM led to a diverse pattern of alterations of other amino acid levels. Indeed, the pairwise comparison revealed a significant decrease in phenylalanine and lysine and an increase in tryptophan, tyrosine and proline plasma levels in patients three months after the intervention. Interestingly, the relative concentrations of all these amino acids and essential histidine were lowered against control subjects before and three months after the intervention for persisted anabolic processes.

Patients with achalasia demonstrated significantly lower serum creatine levels compared to controls. Avoidance of creatine-rich compounds of diet (particularly meat) due to dysphagia might provide explanation for this observation.

The only biochemical abnormality observed in the patient population was a slight elevation in LDL levels prior to POEM, which normalized afterwards. This suggests that the metabolic changes occurring in these patients are not readily detectable through standard clinical tests, and that additional investigative methods may be necessary to accurately assess the metabolic status of individuals with achalasia.

Analyzing the patients’ bioimpedance scale parameters and body measurements, we observed significant changes in the body composition three months after POEM. In general, POEM led to an increase in body weight in more than 90% of patients, which obviously corresponded to the rise in BMI (Fig. 2). In most individuals, we also noted increased waist and hip circumference (Fig. 2). These alterations are to be ascribed to dietary changes after POEM treatment; patients tolerated the broader range and higher amount of food consistency that was linked to an overall increased energy intake.

**Fig. 4:**
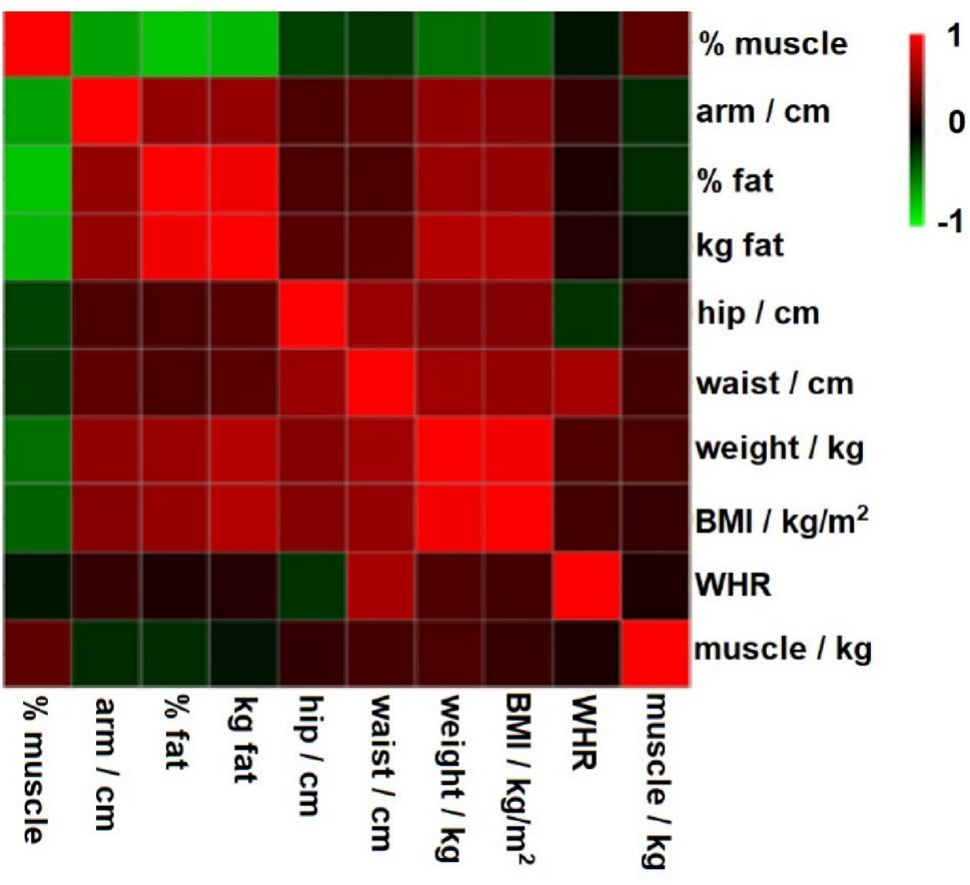
Heatmap for Pearsons’ correlation coefficient to show the connectedness of alterations in bodies’ parameters before-after POEM

Once gained weight, measurable and quantifiable parameters regarding body composition are of great interest as they could provide an interesting insight into the metabolism changes. Indeed, the heatmap (Fig. 4) shows a strong opposite correlation between the increment in relative muscle weight and the increment in absolute and relative fat weight. Moreover, changes in the arm circumference seem to be a good indicator of the increase of the amount of fat during the time of ‘natural’ weight gain, which performed better than the waist-to-hip ratio (showed a much weaker relation to the muscle gain).

**Fig. 5:**
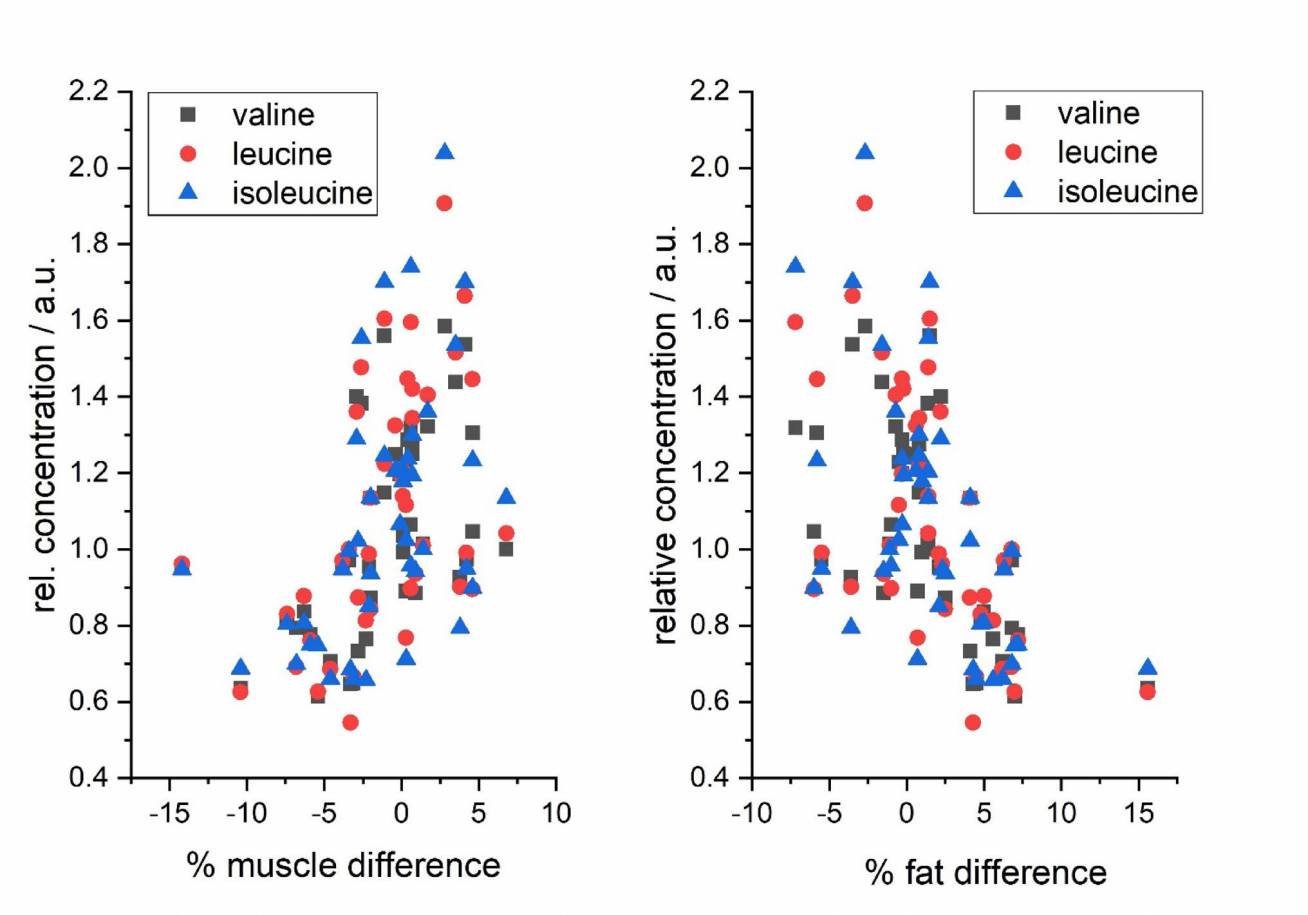
Relation of the pool of circulating BCAAs (leucine, isoleucine and valine) in patients with achalasia before POEM intervention to the changes in body mass composition in three months after the intervention (*p* < 0.005 for all correlations)

Although relatively strong correlations (Fig. 4) were calculated from the data obtained, interestingly, no unique pattern could be recognized regarding the gained amount of absolute or relative fat or muscle gain based on body parameters among individual patients. Although the weight and BMI of typical post-POEM patients increased, we observed differences in the amount of fat or muscle mass gained in individuals after POEM. The question arose whether it is possible to predict the direction of the weight gain – i.e., muscle or fat tissue gain. As the synthesis of proteins in the muscles is essentially dependent on the availability of proteogenic amino acids, we used relative amounts of amino acids plasma levels before POEM (1st visit) as predictors of muscle gain after POEM (2nd visit), calculated as the difference between both time points. As a result, individual plasma levels of BCAAs and their BCKAs (Fig. 5), as well as phenylalanine, tyrosine, glutamine, lysine and histidine, showed significant positive correlations with the relative muscle gain and negative correlation with relative and absolute fat gain (Table 4). All mentioned amino acids behaved very similarly in correlations, which further supports our conclusions. These results did not apply to three amino acids– tryptophan, alanine and proline. The explanation may be derived from the very low representation of these amino acids in muscles against other amino acids (Castell et al., 2009), which, combined with their other metabolic functions, did not yield a significant correlation with increased muscle mass.

The circulating levels of all amino acids (as well as BCKAs) that were marked as related to muscle mass gaining showed similar features: a significant decrease three months after POEM, explained by accelerated protein anabolism/proteosynthesis. Based on this finding, it could be suspected that patients with sufficient amounts of amino acids in the bloodstream are at an advantage in muscle mass gaining compared to those with lower levels in which the gaining of adipose tissue is preferred.

Muscle mass formation was related to the condition before the intervention, as positive correlations with the amino acids pool were only obtained for the concentrations acquired at the 1st visit. No parallel correlations were observed for the 2nd visit (post POEM). This is most probably the consequence of the fact that proteosynthesis is not an instant process and requires an adequate time span. The clinical benefit that could result from our data is the possibility to actively shift the metabolism of patients with yet untreated achalasia towards increased proteosynthesis by providing sufficient amounts of amino acids. Consequent muscle gain instead of fat gain is desirable to limit the long-term negative metabolic consequences of the abundance of fat tissue. Indeed, providing nutritional support to the patient before POEM with the aim to increase the availability of amino acids seems a reasonable approach. Its efficacy should, however, be confirmed in a carefully designed randomized control trial.

Of course, the presented study has its limitations. We did not question the patients’ dietary habits, which surely play an important role in the body’s supply of amino acids and other essential nutrients.

## Conclusion

This study provides a comprehensive analysis of metabolic changes and an in-depth investigation of weight gain in patients with achalasia who underwent peroral endoscopic myotomy. Our approach included NMR metabolomics for the analysis of the changes in metabolic pathways and was combined with standard biochemical blood tests and body composition parameters obtained by the bioimpedance scale to follow up on the consequences of the metabolic shift in 3 months of surveillance. To the best of our knowledge, this is the first study attempting to comprehend the change of metabolic processes in patients with long-term caloric restriction that suddenly gained the ability to have unrestricted oral intake.

Our results indicate that untreated achalasia patients, typically characterized by reduced nutrient intake, show turnover in energy metabolism as their metabolic profile suggested lowered glucose reservoirs, decreasing glycolysis and diminished body’s ability to fuel gluconeogenesis based on on depletion of the major gluconeogenetic precursors such as lactate and amino acids alanine and glutamine. These energy processes were substituted by enhanced ketogenesis. After POEM interventions, and likewise bigger nutrient intake, metabolism improved. Muscle mass increased in patients with sufficient amino acid pool in circulation since the weight gain in other patients was linked rather with the increase in fat tissue. Interestingly, in patients with achalasia, standard biochemical tests failed to detect the metabolic alterations, indicating that conventional diagnostics may not adequately reflect their metabolic condition.

The data were obtained in achalasia patients. We suggest that metabolic changes during fasting/inadequate caloric intake and restoration of normal eating could also be investigated in the general population. We suggest that the metabolism of achalasia patients (yet untreated) should be further investigated as their state of undernutrition could not be imitated in healthy population. We consider our data important not only from the biochemical perspective but also from the standpoint of direct clinical impact. Indeed, nutritional and dietary interventions could be guided based on our results, attempting to shift the metabolic pathways towards proteosynthesis and muscle gain instead of fat gain with potentially undesirable consequences.

## Data Availability

No datasets were generated or analysed during the current study.
